# 24-hour holter electrocardiography in the evaluation of French Bulldogs before and after rhinoplasty and staphylectomy procedures for correction of anatomical abnormalities of brachycephalic syndrome

**DOI:** 10.1007/s11259-026-11185-5

**Published:** 2026-03-28

**Authors:** Francesca Lopes Zibetti, Vitória Ramos de Freitas, Andrielly Wittzorecki Zaikowski, Pamela Regina Pimenta Busato, Esther Ayomikun Oluwagbemiga-Dada, Patrícia Silva Vives, Bernardo dos Santos Vaz, Tatiana Champion, Paula Priscila Correia Costa, Marlete Brum Cleff

**Affiliations:** 1 Department of Veterinary Medicine, Federal University of Pelotas, Pelotas, Rio Grande do Sul Brazil; 2 Veterinary School, Federal University of Pelotas, Pelotas, Rio Grande do Sul Brazil; 3 Department of Veterinary Medicine, Federal University of Fronteira Sul, Realeza, Paraná Brazil; 4 Department of Animal Production and Health, Federal University of Technology, Akure, Ondo State Nigeria; 5 Sul-Rio-Grandense Federal Institute of Education, Science and Technology, Pelotas, Rio Grande do Sul Brazil

**Keywords:** Brachycephalic, Cardiology, Dogs, Pulmonology

## Abstract

The aim of this study was to evaluate changes in 24-hour Holter electrocardiography parameters in French Bulldogs with nostril stenosis and elongated soft palate before and after corrective surgeries. Nine French Bulldogs diagnosed with brachycephalic syndrome (BOAS) were evaluated. Clinical evaluation involved a detailed anamnesis guided by literature-reported clinical signs, followed by a thorough physical examination of both general and respiratory health and a complete screening evaluation and 24-hour Holter electrocardiography. Also, dogs older than 6 years also underwent echocardiography. Assessments were conducted at two time points: initially (pre-surgery) and again between 6 and 12 months postoperatively. After evaluation, animals who were confirmed to have stenotic nares and elongated soft palates and deemed systemically healthy were approved for surgical correction. Examinations performed six months after the rhinoplasty and staphylectomy procedures indicated a persistent high parasympathetic predominance. This study reveals that, even after corrective surgeries for nostril stenosis and elongated soft palate, French Bulldogs with brachycephalic syndrome continue to exhibit a high degree of parasympathetic predominance, which is an important contribution to the limited body of research comparing pre- and post-operative 24-hour Holter electrocardiographic findings in this breed.

## Introduction

The Holter electrocardiogram (ECG) is a complementary test that enables monitoring of autonomic balance and detection of transient cardiac rhythm disturbances over 24 h (Tarelho et al. 2023). It allows analysis of heart rate and rhythm during daily activities, such as rest and exercise, and during clinical episodes (Mavropoulou et al. 2021), making it a valuable tool for patient follow-up and for informing the diagnosis and treatment of cardiac disorders. Dogs with brachycephalic obstructive airway syndrome (BOAS) have increased resistance to airflow due to upper airway obstruction, which can negatively affect various body systems. In the cardiovascular system, this obstruction can lead to changes in rhythm and heart rate of affected dogs (Canola et al. 2018; Tarelho et al. 2023). This occurs due to the high parasympathetic predominance in brachycephalic breeds, which have greater vasovagal tone (Dias et al. 2016).

Diagnosis of BOAS is based on anamnesis, physical examination, and imaging studies. Radiography, endoscopy, and computed tomography play an important role in evaluating static and dynamic obstructions of the respiratory tract (Krainer and Dupré 2022). In addition, the whole-body barometric plethysmography method can be used to objectively assess respiratory function (Liu et al. 2016). The recommended therapeutic approach for correcting the obstructive changes associated with BOAS is surgery, with the goal of improving normal respiratory function and improving the patient’s quality of life (Dupré and Heidenrich 2016). Staphylectomy is performed to treat elongation of the soft palate and involves resecting the excess caudal palatine tissue (Ekenstedt et al. 2020). Likewise, rhinoplasty is performed to correct stenotic nares by wedge resection of the nasal ala, followed by primary closure (Krainer and Dupré 2022). These procedures aim to improve the respiratory status of brachycephalic dogs with BOAS, leading to the hypothesis that cardiovascular conditions associated with airway obstruction may also improve post-surgically. Therefore, this study aims to evaluate changes in 24-hour Holter electrocardiographic parameters in French Bulldogs undergoing rhinoplasty and staphylectomy to better understand the impact of BOAS on the cardiovascular system and the potential influence of surgical correction on these parameters.

## Materials and methods

### Study design

Nine French Bulldogs, regardless of sex, age, or reproductive status, were included in this study. All surgical (rhinoplasty and staphylectomy) and anesthetic procedures were performed at the Veterinary Clinical Hospital of the Federal University of Pelotas (HCV-UFPel) by a single board-certified surgeon and a professional anesthesiologist, following updated protocols for brachycephalic breeds. The study was approved by the UFPel Ethics Committee (CEUA No. 23110.017632/2022-84).

Clinical evaluation of dogs diagnosed with BOAS involved a detailed anamnesis guided by literature-reported clinical signs, followed by a thorough physical examination of both general and respiratory health. Assessments were conducted at two time points: initially (pre-surgery) and again between 6 and 12 months postoperatively. The degree of nostril stenosis was classified according to Liu et al. (2016) as follows: no stenosis (nostrils wide open); mild stenosis (nostrils slightly narrowed, without contact between the lateral and medial walls); moderate stenosis (the lateral wall contacts the medial wall dorsally, leaving the nostrils patent only ventrally); and severe stenosis (nostrils nearly closed, typically associated with mouth breathing). Soft palate elongation was assessed intraoperatively under general anesthesia by evaluating whether the posterior displacement of the soft palate extended beyond the tip of the epiglottis. The presence or absence of laryngeal collapse was assessed during airway evaluation, although its severity was not graded.

Dogs showing clinical signs of BOAS underwent a complete screening evaluation and 24-hour Holter electrocardiography. Animals confirmed to have stenotic nares and elongated soft palates and deemed systemically healthy were approved for surgical correction. Dogs with pre-existing cardiac disease were excluded.

## Data collection

The Holter ECG was performed on the patients at two time points: first, during the week before surgery, and then six to twelve months after rhinoplasty and staphylectomy. For the Holter ECG, the CardioLight 4-channel device by Cardios^®^ was used. Each lead was then attached as follows: white (level of the manubrium), red (level of the xiphoid process), green (mid-thoracic line at the level of the right 5th ICS), and black (mid-thoracic line at the level of the left 5th ICS). After 24 h, the Holter device was removed, and the data card was downloaded to a computer for analysis using CardioSmart^®^ Professional CS 540 software. All examinations were interpreted by the same veterinary cardiologist.

For the evaluation of the examinations and preparation of the reports, atrioventricular blocks were assessed individually and manually by the veterinary cardiology professional. First-degree atrioventricular blocks were not evaluated due to the need to manually measure all PR intervals over the 24-hour period. For the assessment of the ST segment, an elevation was defined as an ST segment greater than 0.15 mV, and a depression was defined as an ST segment less than − 0.2 mV, as described by Tilley and Goodwin (2002). Additionally, for the heart rate evaluation, artifacts and arrhythmias were excluded.

The dogs were fasted for 8 to 12 h before surgery. The patients underwent a standardized anesthetic protocol. Orotracheal intubation was performed, and, at this time, laryngoscopy was also conducted to assess for the presence of an elongated soft palate. Staphylectomy, performed to correct soft palate elongation, was followed by rhinoplasty to address nostril stenosis. Both procedures were carried out as described by Fossum (2013). The patients were clinically evaluated starting 6 months after the surgical procedures to monitor the progression and clinical status related to BOAS, at which time the Holter equipment was reapplied.

### Statistical analysis

The data were analyzed descriptively and statistically using GraphPad Prism^®^ software. For numerical variables, normality was assessed using the Shapiro-Wilk test. Based on these results, parametric data were subjected to the paired t-test, while non-parametric data were analyzed using the Wilcoxon test.

## Results

The minimum and mean heart rates, SDNN, SDANN, rMSSD and sinus arrest episodes were compared using the paired t-test, whereas the maximum heart rate, atrioventricular block episodes, ST-segment elevation episodes, and ST-segment depression episodes were analyzed using the Wilcoxon signed-rank test.

Between March 2023 and December 2024, a total of 66 French Bulldogs were evaluated. A total of 20 underwent surgical procedures, and 9 returned for follow-up six months later. Among the nine dogs that completed both pre- and postoperative Holter examinations, five were female (55.5%) and four were male (44.5%). At the time of their first consultation, their ages ranged from 10 months to 6 years, with the majority being adults (77.7%). Regarding reproductive status, five dogs were neutered (55.5%), and four were intact (44.5%). Owners reported increased sleeping and reduced activity in their dogs while wearing the Holter device, both before and after the surgical procedures. Consequently, most dogs spent the majority of the monitoring period either sleeping or engaged in low activity. Clinical improvement in snoring, exercise intolerance, and tachypnea was observed following rhinoplasty and staphylectomy.

The predominant baseline rhythm in all dogs, both pre- and postoperatively, was sinus arrhythmia. Numerous episodes of sinus arrest (sinus pauses) were also observed and a minor increase in the average duration of the longest pause, from 2.9 to 3.0 s (*p* > 0.05). Most pause episodes were concentrated during the night and early morning hours, coinciding with the dogs’ sleep periods. The atrioventricular (AV) blocks observed in these cases were classified electrocardiographically as a Mobitz type II pattern (Table 1). One outlier dog accounted for AV blocks for 967 episodes before and 120 after the surgical procedures. Second-degree atrioventricular block was classified as Mobitz type II based on a constant PR interval in conducted beats with sudden non-conducted P waves and no evidence of progressive PR prolongation suggestive of a Wenckebach pattern. Furthermore, Table 1 presents the results of ST-segment elevation episodes and ST-segment depression episodes. It is important to point out that no dogs exhibited syncope, presyncope, weakness, or other clinical signs potentially attributable to arrhythmias during the Holter monitoring period, either pre- or postoperatively.

**Table 1:** Sinus arrest, atrioventricular block, ST segment elevation and depression (number of episodes in Holter ECG of French Bulldogs before and after rhinoplasty and staphylectomy)

Variable	Before	After
*Sinus Arrest*	20319.67 ± 7347.45	23297.44 ± 6791.80
Atrioventricular block	5 (0–967)	1 (0–120)
ST segment elevation	0 (0–29)	2 (0–46)
ST segment depression	0 (0–28)	0 (0–17)

Values are presented as median and min-max, except for sinus arrest (expressed as mean ± standard deviation)

The minimum, maximum, and mean heart rates, as well as the episodes of sinus tachycardia and bradycardia, are presented in Table 2. All time-domain heart rate variability (HRV) parameters (SDNN - standard deviation of all normal RR intervals; SDANN - standard deviation of the averages of normal RR intervals every 5 min; rMSSD - root mean square of successive differences between adjacent normal RR intervals) showed some increase following rhinoplasty and staphylectomy (Table 2).

**Table 2:** Heart rate variability (time domain) and minimum, mean, and maximum heart rates in French Bulldogs before and after rhinoplasty and staphylectomy

Variable	Before	After
Max HR (bpm)	247 (215–250)	250 (242–250)
Min HR (bpm)	44 ± 6.84	43 ± 5.45
HR Mean (bpm)	93.3 ± 12.25	93.1 ± 10.89
SDNN (ms)	282.7 ± 49.09	296.7 ± 50.52
SDANN (ms)	159.2 ± 54.29	187.3 ± 51.50
rMSSD (ms)	129.2 ± 46.21^a^	165.4 ± 58.66^b^

Values are presented as mean ± standard deviation, except for max HR (bpm) (expressed as median and min-max)

* HR* heart rate, *SDNN* standard deviation of all normal RR intervals, *SDANN *standard deviation of the averages of normal RR intervals every 5 min, *rMSSD* root mean square of successive differences between adjacent normal RR intervals. Different lowercase superscript letters indicate significant differences between groups (*p* < 0.05).

## Discussion

Sinus arrhythmia was the predominant baseline rhythm in all dogs at both evaluated time points. This finding is consistent with the high prevalence of sinus arrhythmia in brachycephalic breeds, which is considered a normal rhythm associated with increased vagal tone (Canola et al. 2018; Silva et al. 2022). Notably, some studies suggest that the incidence of sinus arrhythmia may decrease after surgical procedures in these breeds (Filho et al. 2020).

All evaluated dogs exhibited multiple episodes of sinus arrest at both time points. The average number of episodes increased after corrective surgery (from 20.319.67 to 23.297.44), and the average duration of the longest pause also showed a slight increase (from 2.9 s to 3.0 s). This contrasts with findings by Filho et al. (2020), who reported a reduction in sinus arrest episodes following rhinoplasty. Sinus arrest is defined by an RR interval lasting at least twice as long as the previous one and results from delayed impulse generation by the sinoatrial node (Tilley and Goodwin 2002; Silva et al. 2022), typically due to increased vagal tone (Filho et al. 2020). Therefore, although the mean longest sinus pause was approximately 3.0 s, pauses of this duration may occur during periods of high vagal tone, especially during rest or sleep. Therefore, in clinically asymptomatic dogs, this finding alone does not necessarily indicate pathological sinus node dysfunction.

After rhinoplasty and staphylectomy surgeries, a reduction in episodes of atrioventricular block (AVB) was observed. Most AVBs detected were classified electrocardiographically as a Mobitz type II pattern, characterized by an interruption in conduction in the His or infra-His region. This type of AVB can occur in conditions of increased vagal tone, often associated with chronic respiratory diseases (Santilli et al. 2018). However, although this pattern was identified based on electrocardiographic criteria, the ambulatory nature of Holter recordings does not allow complete exclusion of vagally mediated AV conduction disturbances. Increased parasympathetic tone, particularly during rest or sleep, may produce AV block patterns that mimic Mobitz II. Therefore, the findings should be interpreted in the context of functional autonomic modulation rather than definitive intrinsic conduction system disease.

When assessing ST segment changes, whether elevation or depression, no significant change in the number of episodes was observed pre- and postoperatively. Elevation or depression of the ST segment may indicate myocardial tissue hypoxia (Santilli et al. 2018).

The occurrences of sinus tachycardia and sinus bradycardia increased in the evaluated dogs following rhinoplasty and staphylectomy procedures. Sinus tachycardia is common in adult dogs during moments of excitement or exercise. It can also arise from increased automaticity of normal pacemaker cells, shifts in the site of atrial depolarization origin (within the sinoatrial node), or the presence of a reentry microcircuit (Santilli et al. 2018). Additionally, reduced oxygen circulation caused by hypoxia is detected by carotid chemoreceptors, triggering a compensatory sinus tachycardia response (Rey et al. 2007). Increased vagal tone in cases of chronic respiratory diseases may lead to bradyarrhythmias such as sinus bradycardia (Filho et al. 2020). Brachycephalic dogs exhibit more pronounced sinus arrhythmia compared to other breeds, particularly during sleep or deep breathing (Santilli et al. 2018).

All time-domain heart rate variability (HRV) parameters increased following the surgical procedures. Brachycephalic breeds typically exhibit prolonged inspiration and a heightened respiratory influence on the autonomic nervous system, which can affect HRV (Fernandes et al. 2024). The vagus nerve modulates parasympathetic cardiac activity, producing oscillations in the intervals between heartbeats—referred to as vagally mediated HRV. Increased HRV reflects parasympathetic predominance (Han et al. 2021). Time-domain HRV assesses variations in RR intervals and provides insight into parasympathetic tone (Santilli et al. 2018). A reduction in both SDNN and rMSSD would suggest diminished parasympathetic activity (Filho et al. 2020); however, this was not observed in the evaluated dogs. In contrast, Filho et al. (2020), using a five-minute Holter ECG, reported a decrease in SDNN and rMSSD following surgical intervention.

Although HRV indices and rhythm analysis indicated a relative increase in parasympathetic markers after surgery, this finding should be interpreted with caution. Increased parasympathetic modulation does not necessarily indicate pathological autonomic dysfunction and may instead reflect physiological adaptation following surgical correction. Improved comfort, reduced respiratory effort, deeper sleep, and long-term autonomic remodeling after relief of chronic upper airway obstruction may all contribute to this autonomic profile (Bonaduce et al. 1998; Shaffer and Ginsberg 2017). Importantly, these electrocardiographic changes occurred alongside postoperative clinical improvement, supporting the interpretation of favorable autonomic rebalancing rather than deterioration.

Study limitations included sample size and the duration of postoperative follow-up. Future studies involving a larger number of animals and longer follow-up periods are needed to confirm these findings and further explore the relationship between cardiac alterations and BOAS. Furthermore, owner-reported reductions in activity level and increased sleep during Holter monitoring may have influenced autonomic tone, favoring parasympathetic predominance. This may have contributed to changes in HRV indices as well as to the frequency of sinus arrest and bradyarrhythmias. Daytime and nighttime recordings were not analyzed separately, which represents a limitation and should be considered when interpreting these results. Postoperative Holter evaluations were performed between 6 and 12 months after surgery to assess late cardiovascular adaptation. However, autonomic modulation may continue to evolve during this period, and grouping these time points may have introduced variability. Therefore, the findings should be interpreted as reflecting general late postoperative trends rather than discrete time-dependent changes.

## Data Availability

The datasets generated and analyzed during the current study are not publicly available but are available from the corresponding author upon reasonable request.
